# Evaluation of forces on muscles and intervertebral joints during active kyphosis-deepening exercises in the dynamic individual stimulation and control for spine device (DISC4SPINE, D4S): a modeling approach—proof of concept study

**DOI:** 10.3389/fspor.2025.1642246

**Published:** 2025-11-06

**Authors:** Tomasz Szurmik, Karol Bibrowicz, Katarzyna Nowakowska-Lipiec, Hanna Zadoń, Robert Michnik, Andrzej Waldemar Mitas

**Affiliations:** 1 Faculty of Arts and Educational Science, University of Silesia, Cieszyn, Poland; 2 Science and Research Center of Body Posture, Kazimiera Milanowska College of Education and Therapy, Poznan, Poland; 3 Faculty of Biomedical Engineering, Silesian University of Technology, Zabrze, Poland

**Keywords:** scoliosis, muscles, intervertebral joints, ANYBODY, PRESSIO, DISC4SPINE

## Abstract

**Introduction:**

In the conservative treatment of scoliosis, it is important to monitor the response of the muscles and the forces acting on the intervertebral joints during the recommended therapy. This study aimed to evaluate the forces exerted by the limbs and intervertebral joints, as well as the forces of selected muscles on both sides of the back, during active spinal kyphosis exercises in the supported kneeling position used in the PRESSIO method.

**Methods:**

An experimental biomechanical investigation was conducted using a prototype of the DISC4SPINE system. One healthy subject was examined. During the exercise, contact forces acting on the surfaces of the hands and knees, as well as forces generated by the system's resistance elements acting on the subject's body, were recorded. The kinematic parameters of the movement were documented using a video camera. The collected measurement data was used to inform simulations conducted within the ANYBODY modelling system environment, employing the FreePosture whole-body model. Two positions were modelled: position 0 represented the resting state with no exercise or active interaction with the system's heads, and position 1 represented kyphotic movements of the spine with simultaneous interaction with the system's resistance elements.

**Results:**

The simulation results showed a significant increase in mean force values acting on the upper and lower extremities in the active kyphotic position compared to position 0.

**Discussion:**

The supported kneeling position employed in the PRESSIO method is characterised by reduced force exerted along the long axis of the spine, creating favourable conditions for correction. An increase in the average values of intervertebral forces was also observed in position 1 compared to position 0. Furthermore, active kyphosis of the spine caused an increase in muscle activity in the back extensor muscles (Erector Spinae, ES).

## 1 Introduction

Scoliosis is a three-dimensional deformity of the spinal column that results in changes in the alignment of the trunk and pain (1, 2). This condition has the potential to manifest during early childhood, affecting 1%–12% of the general population of children and adolescents (3). The most prevalent form of the condition is idiopathic adolescent scoliosis (AIS), the etiology of which remains to be fully elucidated (4). The management of patients afflicted with scoliosis poses a considerable challenge for physiotherapists and physicians alike, primarily due to the indeterminate nature of the underlying cause. In the absence of a discernible etiology, a predominant approach entails the implementation of symptomatic management. A three-pronged therapeutic approach is recommended, encompassing both conservative and surgical methodologies, as well as the utilization of corsets (2, 5). A plethora of conservative treatment methods are employed in the management of scoliosis, including Lyon, Schroth, SEAS, FITS, BSPTS, DOBOMED and SIDESHIFT. As indicated in the works of Dimitrijevic et al. (6), Romano et al. (7), and Berdishevsky et al. (8).

Certain methodologies also employ devices or instruments for three-plane correction with pressure elements. As demonstrated in the FED method (9) pressure can be induced by external forces. Conversely, the PRESSIO method (10, 11) utilizes pressure that is induced by the patient's own force. In the planned therapy, it is imperative to take into account the principles of effective training, and the pressure force should be skillfully dosed. In numerous methods, the knowledge and experience of the physiotherapist serve as the primary determinants of the training selected. A distinct issue pertains to the monitoring of muscle response to the recommended therapy, which has the potential to yield more favorable outcomes in terms of spinal correction. A significant challenge exists in the measurement of muscle response *in vivo* during exercise, particularly in the deep layer, i.e., the spinal muscles. These muscles are of paramount importance in the therapeutic management of scoliosis. However, measuring these muscles poses a substantial ethical challenge. In the therapeutic management of patients with scoliosis, it is imperative to meticulously monitor the muscular activity on both the concave and convex sides of the curvature. This is due to the observed disparities in their respective alterations, which have the potential to serve as either a causative agent or a consequence of the underlying disease (12). The individualized observation of muscle response on both the concave and convex sides of the curvature for each patient is imperative for the planning of safe and effective therapy. In recent years, mathematical modeling methods of the musculoskeletal system have become increasingly popular for the assessment of joint reaction forces and estimation of muscle force activity. One commercial environment that allows for the analysis of internal forces without the need for direct intervention in the patient's body is the ANYBODY Modeling System. This system has been utilized in diagnostic studies on the reconstruction of a 3D musculoskeletal model of the thoracolumbar spine based on digital radiographic images obtained with EOS (13, 14) and the prediction of scoliosis progression (15). A review of the literature reveals the existence of studies on the musculoskeletal dynamics of patients diagnosed with adolescent idiopathic scoliosis (AIS). As indicated by Bassani et al. (16) and Bibrowicz et al. (17), there is a discrepancy in muscle parameters on the concave and convex sides of patients with scoliosis (18). The utilization of Anybody modeling has been examined in the context of the musculoskeletal system of the upper limb (19) and the lumbar-pelvic complex (20–22). However, a paucity of literature exists regarding measurements of muscle responses to therapeutic exercises. The acquisition of such knowledge has the potential to enhance the efficacy of conservative interventions and individualized exercise regimens tailored to a specific patient.

The objective of this study was to assess the forces exerted on the upper and lower limbs, intervertebral joints, and the forces of selected muscles on the left and right sides of the back during active kyphosis exercises of the spine in the supported kneeling position, as utilized in the PRESSIO method, as proof of concept study**.** The exercises were executed using the DISC4SPINE System. Experimental and model tests were carried out on a healthy person without diagnosed scoliosis, and the head was set to mimic the thoracic, right-sided curvature of the spine.

## 2 Methods

### 2.1 Research methodology

The objective of this study was to determine the loads occurring in the human musculoskeletal system during exercise in the kneeling-supported position. To this end, simulations were carried out in the ANYBODY Modeling System environment (ANYBODY Technology Inc., Aalborg, Denmark). Prior to initiating the simulations, it was imperative to collect input data for the model. This entailed measuring the kinematics of the exercises and the ground reaction forces, their point of application, and the forces on the resistance heads. The measurements were conducted in 2,021 as part of the project “DISC4SPINE (D4S): Dynamic Individual Stimulation and Control for Spine and Posture Interactive Rehabilitation”, which received funding from the European Union under the reference POIR.04.01.02-00-0082/17.

### 2.2 Experimental studies

**Figure 1:**
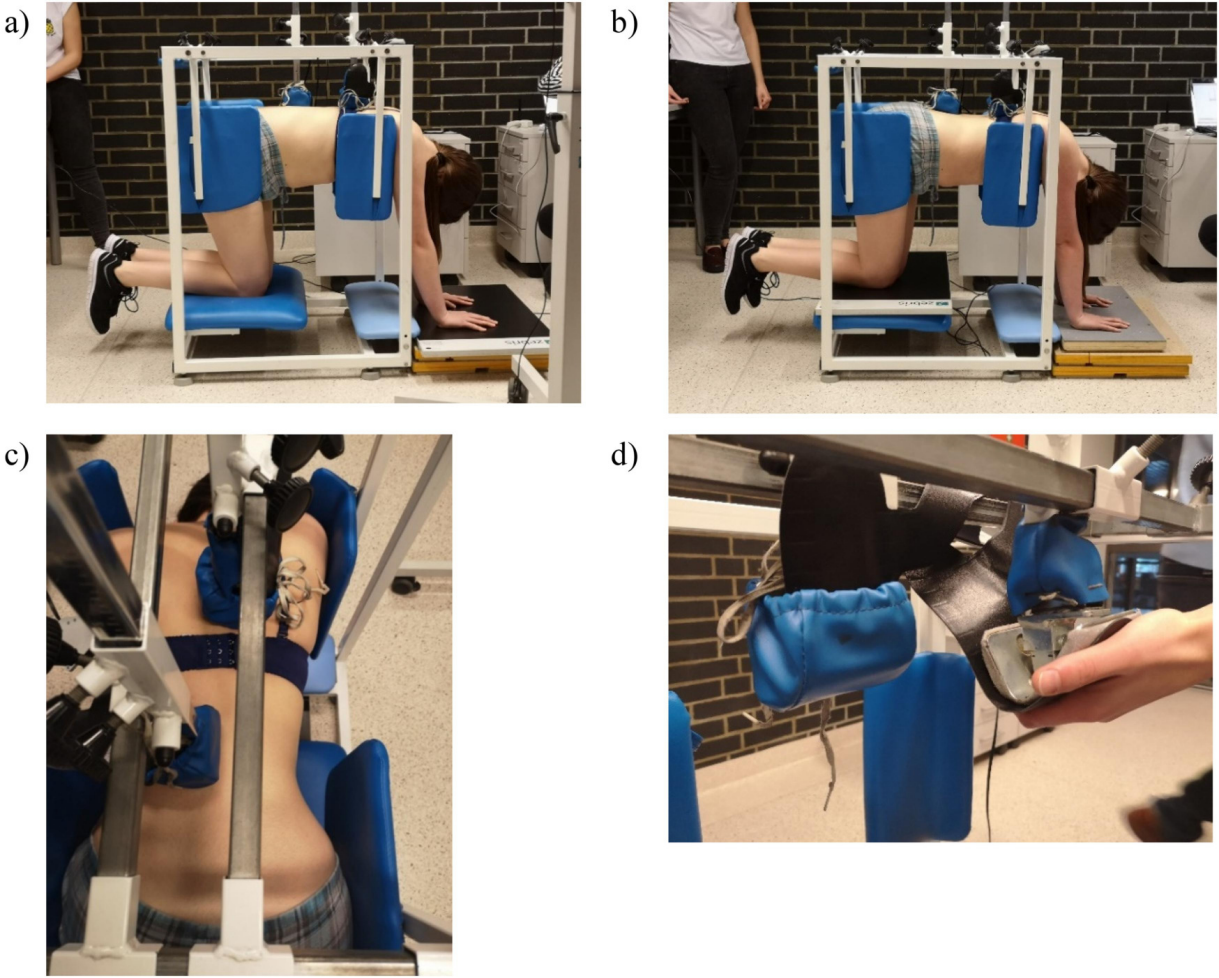
Exercises of the participant in the initial prototype of the DISC4SPINE system: **(a)** Kneeling-supported position with the measurement platform under the hands, with therapeutic heads interacting with the spine and ground reaction forces of the upper limbs recorded. **(b)** Kneeling-supported position with the measurement platform under the knees, with ground reaction forces of the lower limbs recorded. **(c)** Top view showing the arrangement of the therapeutic heads on the spine. **(d)** Close-up illustrating the placement of Medilogic measuring inserts on the heads.

Biomechanical experimental research was carried out on a preliminary prototype of the DISC4SPINE system. The research focused on the kneeling-supported position test module, as illustrated in Figure 1. The study's subject was a healthy woman in her 20s with a height and weight of 171 cm and 66 kg, as a physiological basis for a future physiopathology study - proof of concept study. The subject was found to be free of musculoskeletal disorders and other diseases. The measurements were obtained under the supervision of a seasoned physiotherapist, who provided detailed instructions on the proper execution of the exercise. During the DISC4SPINE System exercise, the subject assumed a supported kneeling position, with stabilization of the shoulder and hip girdle provided by special locks. The superior aspect of the device was located between the sixth and eighth vertebrae on the right side of the thoracic spine, at a distance of approximately 1.5 centimeters from the spinous processes, within the upper range of possible kyphotic movement. The head positioning utilized in this case was specifically designed for patients exhibiting right-sided thoracic curvature. The patient was instructed to perform an exercise known as “cat's back”, during which the head was positioned in contact with the spine. This maneuver was intended to induce derotation, correction, and redression of the spine. The experiment was replicated on three separate occasions, and the mean value of the results was calculated. The study was approved by the Bioethics Committee (No. 3/2019) operating at the J. Kukuczka University of Physical Education in Katowice, in accordance with the Declaration of Helsinki.

The values of forces acting on specific points of the human body during exercise in the kneeling-supported position were measured in a module for exercise. This was accomplished with the aid of biomechanical measuring apparatus. Pressure forces under the hands and knees were measured using a Zerbis FDM-S dynamometer platform (ZebrisMedical GmbH, Isny, Germany) equipped with capacitive force sensors (2,560 sensors, recording frequency 200 Hz, measurement accuracy ±5%). Medilogic measuring inserts (T&T medilogicMedizintechnik GmbH, Schönefeld, Germany) were utilized to assess the forces with which resistive elements interact with the human body during exercise. Each insert contains 130 capacitive force sensors with a recording frequency of 200 Hz; measurement accuracy ±5%. The inserts were meticulously positioned between the metal component of the head and the spongy sheath (Figure 1d). The kinematic parameters of movement during the exercise were meticulously recorded using a SONY HDR-CX625 model video camera (sampling rate: 25 Hz, Full HD: 1,920 × 1,080 resolution videos) positioned perpendicular to the sagittal plane of the subject, who was situated within the DISC4SPINE system module. Synchronization between force data (200 Hz) and video recordings (25 Hz) was performed manually using a predefined event (a tap on the force platform), visible in both datasets. The video-derived kinematic data were then interpolated to match the sampling rate of the force data, enabling their coherent integration for modeling purposes.

**Figure 2:**
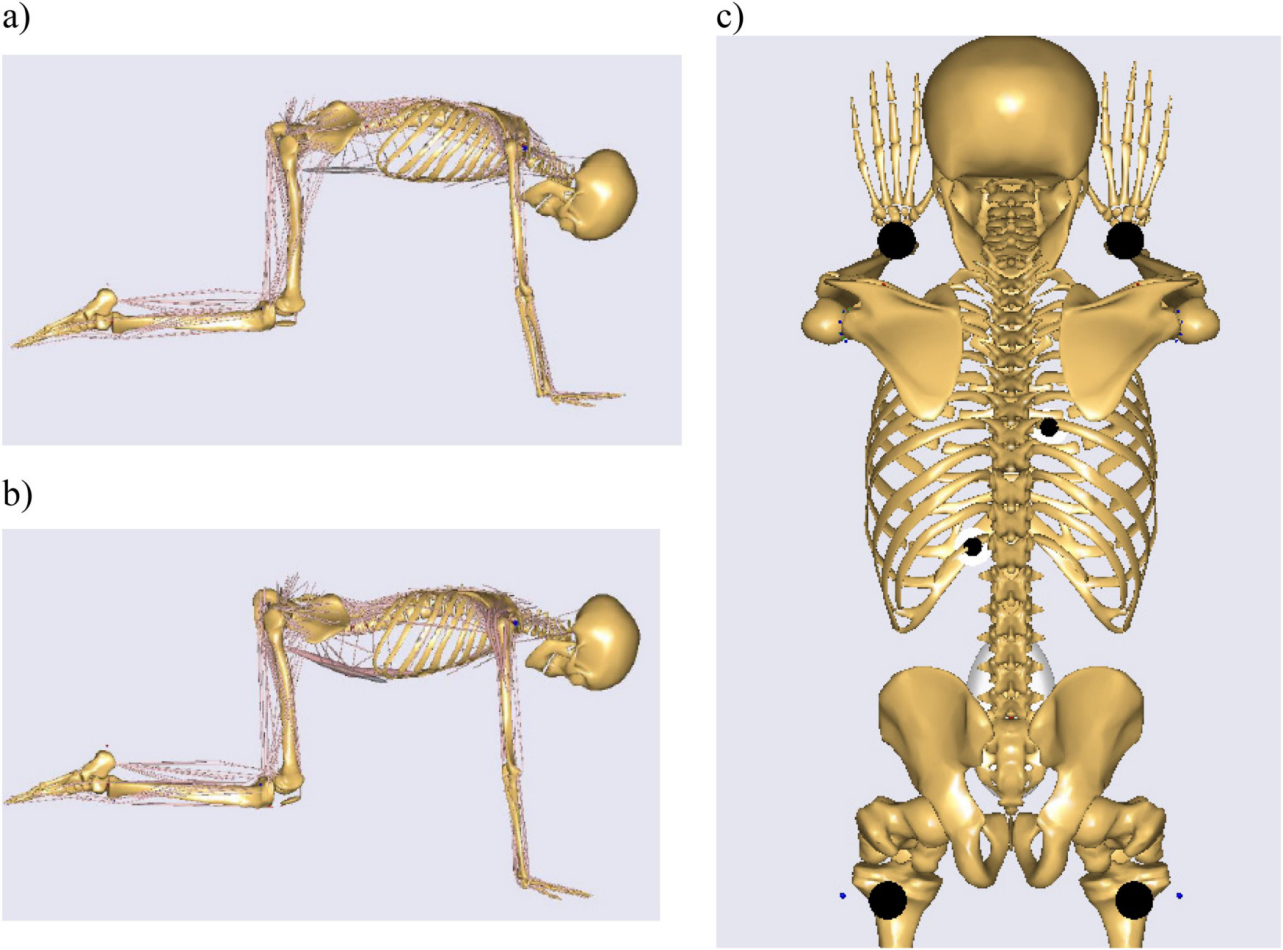
The following positions have been modeled in the AnyBody system: **(a)** Position 1, **(b)** Position 0, **(c)** location of force application.

A comprehensive set of measurement data was collected for the entirety of the movement/exercise, while two positions were selected for modeling in the Anybody environment:

- Position 0: Position in the DISC4SPINE module without exercise and head interaction (Figure 2a).
- Position 1: The first position is characterized by active kyphotization of spine and pressure on the head, within the DISC4SPINE module (Figure 2b). For each position, the same time instant was selected for which results were exported.

### 2.3 Model tests

The measurement data that had been collected was utilized as the input for running simulations in the ANYBODY Modeling System environment. The FREEPOSTURE whole human model was utilized in this study. The human musculoskeletal system model consists of 69 rigid solids, representing bones, which are connected by kinematic pairs, representing joints. The musculoskeletal system is comprised of approximately 1,000 muscle actions. Muscles are conceptualized as elastic-damping elements that are affixed to body segments, thereby facilitating movement. The spinal model under consideration is composed of twelve thoracic vertebrae and a single element representing the thorax. The five lumbar vertebrae are represented by separate segments that are connected by spherical intervertebral joints, which have three degrees of freedom. Finally, the sacrum and the pelvis are modeled as rigidly connected segments (23, 24). The lumbar spine model under consideration encompasses a total of 188 muscle fascicles, which represent the muscles of the abdomen and back. These muscle fascicles include, but are not limited to, the erector spinae, quadratus lumborum, multifidus, transverse abdominis, and rectus abdominis muscles, as well as the abdominal internal and external oblique muscles. The line of muscle action is delineated by the initial and terminal points of attachment, and potentially the intermediate points (via points) through which the muscle passes (23, 25). The spine model also incorporated the IAP intra-abdominal pressure model (21, 22, 26, 27). The arrangement of individual body segments was based on the recorded kinematics. The values of the joint angles and spinal alignment were modeled on the basis of images (corresponding time moments from the recorded video with a camera).

**Figure 3:**
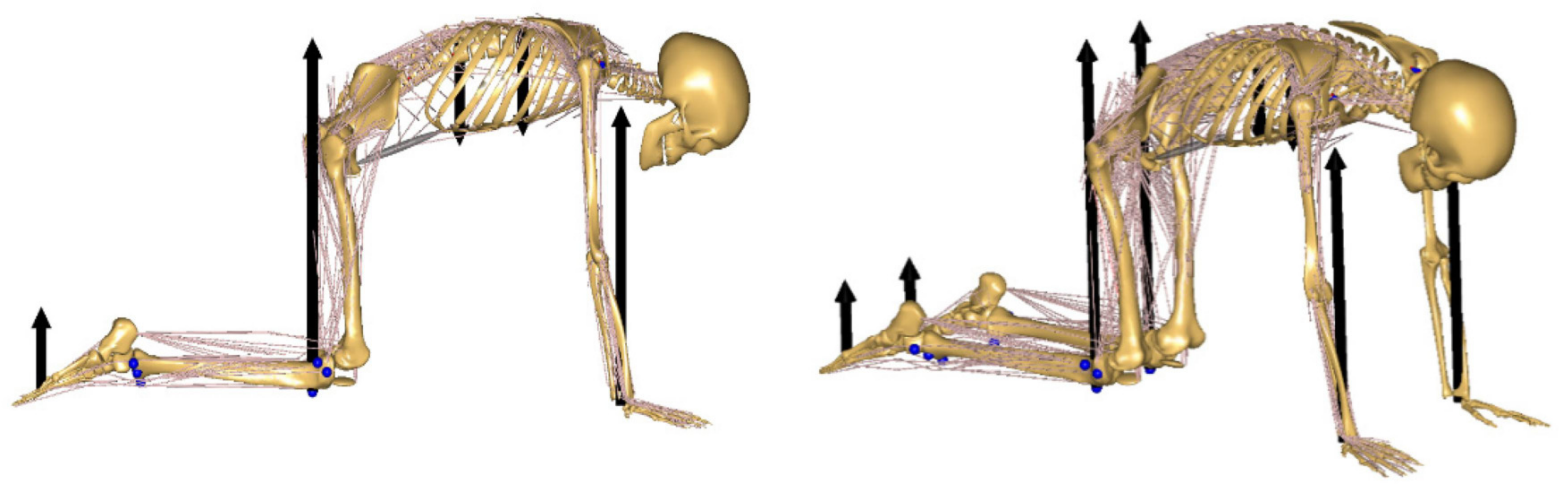
The model of the musculoskeletal system demonstrates the location of force vectors during exercise.

The values of the ground reaction forces of the lower and upper limbs, as well as the values of the head reactions in the model, were entered into the model in the form of force vectors. The values of these vectors were collected during the experimental tests (as the sum of the pressure from all the sensors under each body segment). The force vectors for the lower extremities were applied proximally to the upper tibial epiphysis, while those for the upper extremities were applied distally to the radius bone and the carpal bones. The force vectors of the resistance heads were positioned in close proximity to the transverse processes of the vertebrae. The selection of application sites for the vectors was made on the basis of ongoing studies and color pressure maps, with the most stressed areas being identified for intervention (Figure 2c). The prepared model of the musculoskeletal system, with force vectors positioned during the exercise (Figure 3), is presented herein.

In the ANYBODY Modeling System, a traditional inverse dynamics approach and static optimization are employed to calculate joint force responses and muscle force values when assuming a given body position or movement. This process is performed to minimize muscle recruitment activation. The simulations employed an implicit polynomial optimization criterion (23, 24). The applied model of the musculoskeletal system has been repeatedly validated to evaluate lumbar spine loads and muscle activation during given positions under physiological conditions (13, 14, 23, 27–29, 32). The values of left and right back muscle forces during the performance of active spinal kyphosis in the supported kneeling position used in the PRESSIO method were analyzed. The resulting values of reaction forces in the joints of the lumbar spine and the values of selected muscle forces (Erector Spinae - ES, Multifidi - MF, Obliquus Internus Abdominis - OIA, Psoas Major - PM) in two body positions for the left and right sides were analyzed.

## 3 Results

The values of the average forces in the positions under study demonstrate a clear differentiation between the upper and lower extremities. The measurements revealed that the Position 1 exhibited higher values in comparison to the initial “0” position. In contrast, such clear differences were not observed between the sides of the test. As demonstrated in Table 1, higher average values of forces acting on the lower limbs were observed.

**Table 1:** Mean force values for the upper and lower limbs and under the resistance heads (N) in the studied positions.

Measurement site of pressure forces	Force averages (N)					
	Left side			Right side		
	Position 0	Position1	Difference	Position	Position 1	Difference
Upper limbs	146.5 ± 11.2	178.5 ± 16.3	32	126.5 ± 8.2	179.5 ± 21.9	53
Lower limbs	175.0 ± 13.4	336.0 ± 32.5	161	185.0 ± 15.1	302.0 ± 19.1	117
Resistance head	–	305.8 ± 34.1	305.84	–	113.2 ± 18.6	113.2

**Figure 4:**
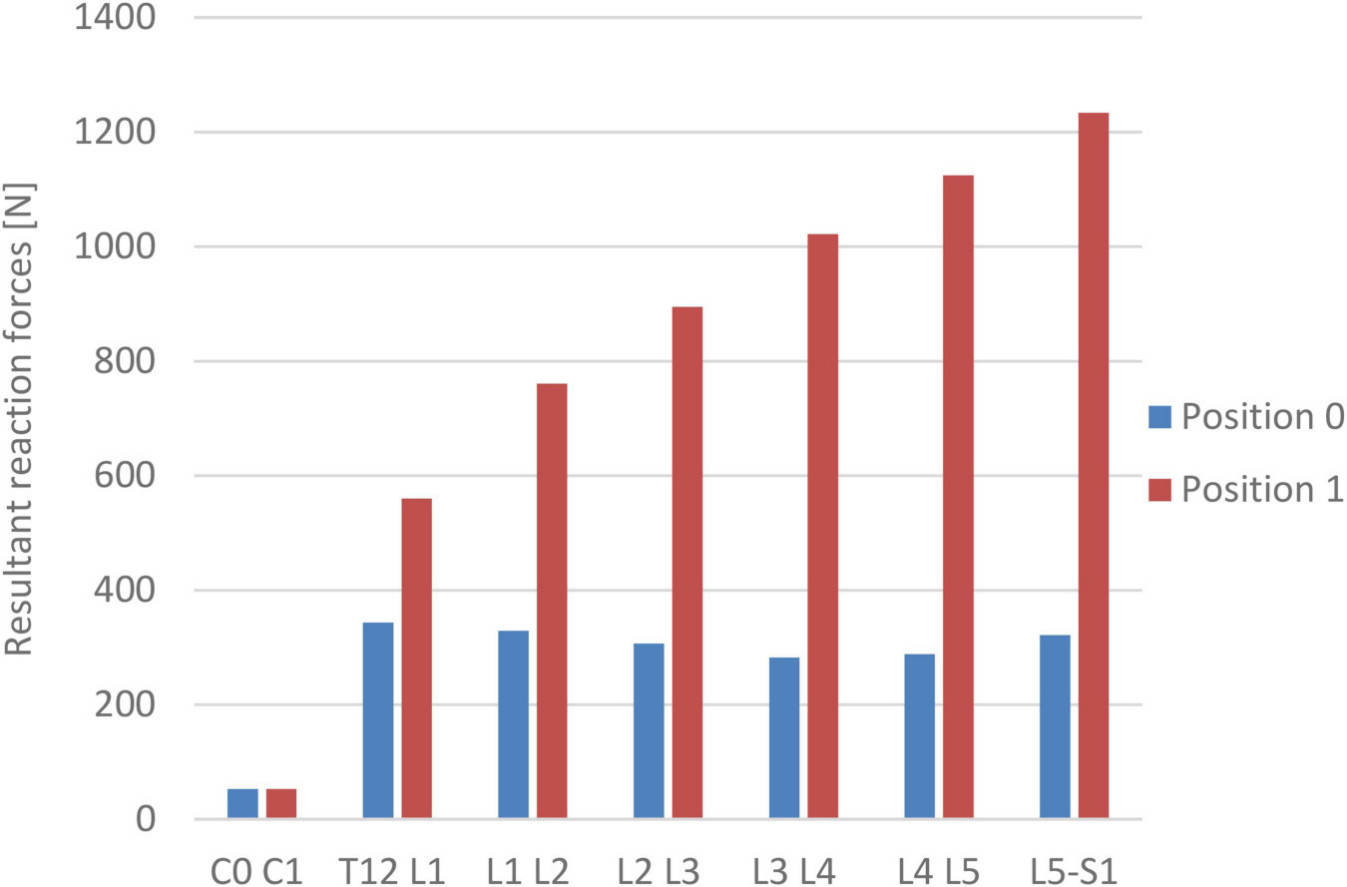
The values of resultant reaction forces in the intervertebral joints.

The values of reaction forces observed during the test demonstrated their variation between the tested positions. The discrepancies were of an escalating nature. The joints at the atlanto-occipital level do not exhibit any differences. However, there is an increase in variation starting from the level of the joints of the thoracic spine (T12-L1) and continuing to the joint at the L5-S1 level. In the “1” position, significantly higher average values of measured forces were identified than in the “0” position (Figure 4).

A subsequent analysis of the results of selected trunk stabilizing muscle forces demonstrated their differential response to alterations in the spine's configuration during active kyphotization in the “1” position and pressure on the resistance heads. A significant body of research has demonstrated that pronounced changes are associated with alterations in the activity of the erector spinae and multifidi muscles. However, the smaller muscles are associated with the obliques internus abdominis and the psoas major muscles (Table 2).

**Table 2:** The force values of selected trunk muscles for positions “0” and “1” during kneeling-supported exercises.

Muscle	Torso muscle force values for positions 0 and 1 during exercises in the kneeling and supporting position (N)					
	Left side			Right side		
	Position 0	Position 1	Difference	Position 0	Position 1	Difference
mm. ErectorSpinae	25.8	417.7	391.8	31.4	461.6	430.2
mm. Multifidi	17.6	144.1	126.6	18.2	114.8	96.7
mm. Obliquus Internus Abdominis	11.6	33.7	22.1	41.5	89.9	48.5
mm. Psoas Major	41.7	14.4	−27.3	16.9	2.1	−14.8

## 4 Discussion

Prevention and conservative treatment of patients with scoliosis are of paramount importance, particularly in the early stages of the disorder. The knowledge and experience of therapists using a variety of physiotherapeutic methods can be supplemented by monitoring and analyzing the relevant parameters of the disorder, both during the actual exercise and before and after treatments.

The objective of this study was to assess the forces exerted on the upper and lower extremities, intervertebral joints, and the forces of selected muscles on the left and right sides of the back during active spinal kyphosis in the supported kneeling position, as utilized in the PRESSIO method as proof of concept study. Our research demonstrated that the active kyphotization position “1” exhibited significantly higher average values of forces for the upper and lower extremities compared to the “0” position. Subsequent analysis failed to identify any significant disparities between the left and right sides. Conversely, elevated mean values of the measured forces for the lower extremities were documented. An augmentation in the values of reaction force within individual intervertebral joints was also observed, commencing from a lack of differences for the joints of the atlanto-occipital level, progressing to elevated variability in the force values of subsequent intervertebral joints of the lumbar spine. The differential response of selected trunk stabilizing muscles to the change in the shape of the spine in the cat's back position “1” and the pressure on the resistance heads was demonstrated.

The most significant changes were observed in the Erector Spinae and Multifidi muscles, while less substantial changes were noted in the Obliquus Internus and Psoas Major muscles. The force values for the Erector Spinae muscles were approximately 400 N higher in the “1” position than in the “0” position, and similarly for, Multifidi the force values were about 100 N higher.

The FED method utilizes the impact of mechanical force on the peak of curvature in patients with scoliosis. A 3-week study employing this method yielded favorable outcomes in the treatment of patients aged 11–17 years with bicondylar scoliosis types I and II, as classified by the King Moe system, with scoliosis angles ranging from 10 to 600 Cobb. The pressure force applied, meticulously calibrated to align with the patient's capabilities, reached a maximum of 100 kilograms. The study patients wore Boston-type orthoses for 21–22 h per day, and the treatment time was 30 min. Concurrently, the patients underwent electrostimulation of the muscles on the convex side of the curvature and were administered warm compresses. A substantial positive change in the angle of torso rotation (ATR) in the thoracic and lumbar spine, as well as the angle of scoliotic deformity, was demonstrated (9). The findings of our research indicated the presence of higher force values for the lower extremities in comparison to the upper extremities, exhibiting a modest predominance for the left side, particularly in the “1” position designated as active kyphotization. The primary distinction between the aforementioned methods lies in the patient's posture during therapy. Specifically, the “PRESSIO” method involves the patient assuming a supported kneeling position, exerting pressure on the resistance heads utilizing the force generated by their own muscles. In the “FED” method, a single curvature arch is targeted during each therapy session. Furthermore, an upright position is employed, with the patient suspended by specialized suspenders and a predetermined value of force dispensed through a computer-controlled mechanical external actuator. The supported kneeling position facilitates the simultaneous differentiation and monitoring of forces in the thoracic and lumbar spine during exercise, contingent on the curvatures and therapeutic objectives.

Bassani et al. (13), proposed a semi-automated software approach to reconstruct a three-dimensional musculoskeletal model of the thoracolumbar spine from digitized radiographic images obtained with EOS. A study was conducted in which intervertebral loads were examined in 38 standing adolescents with mild idiopathic scoliosis. The study then compared these findings with El-Rich studies for those without scoliosis. The ANYBODY software was used in this study. As demonstrated in the works of Bassani et al. (14), and El-Rich (30), the findings concerning the standing position exhibited comparable outcomes to those of El-Rich. The study revealed differential forces acting upon the intervertebral joints during axial compression (Fx) and anterior-posterior compression (Fy). The highest recorded average values were observed at the L1 level, with Fx measuring 557 N and Fy measuring −95 N. Notably, these values did not increase in a gradual manner towards L5, as observed in the El-Rich model.

The company's own research demonstrated a definitive variation in the values of forces at the intervertebral joints between the “0” and “1” positions. The lowest recorded values of reaction forces were observed at a level of 52 N for C0 - C1, with no discernible differences observed between the positions that were studied. The highest values were recorded at the Th 12 -L1 level: 343 N for the “0” position and 560 N for the “1” position. The highest recorded values of reaction forces in the intervertebral joints were observed in the “1” position for the L5-S1 level. Subsequent to normalizing the data to the subject's body weight, the force values for the “0” position were found to be lower than in the Bassani and El-Rich study. However, these values increased in the “1” position. The values of the measured forces exhibited a linear increase in the L5 direction, a finding that aligns with the conclusions of the El-Rich study (30).

Orthopedic provision through the use of various types of rigid as well as dynamic corrective orthoses and corsets is also an indispensable part of scoliosis treatment. Ali et al. published a critical evaluation of the effect of rigid corrective corsets on patients with scoliosis. The study found that the passivity, stiffness, and lack of control of the force they release are key factors in the development of the condition. It is hypothesized that applying excessive pressure to a stiffened spine may exacerbate its condition. The researchers proposed a novel active soft orthosis that allows for the controlled application of corrective forces in conjunction with spinal mobility. The achievement of control over the forces acting on the spine was accomplished by varying the tension in the elastic bands using light actuators comprising low-powered twisted cords. The clamping force is modeled using a belt and pulley contact model and verified by building a custom test stand. The actuator module has been demonstrated to possess the capacity to modulate pressure within the range of 0–6 Kpa, a range that is commensurate with the pressure adjustments employed in rigid corsets, which typically range from 0 to 8 Kpa. Our research endeavors have indicated the manifestation of reduced forces exerted on the intervertebral joints. This observation is partially corroborated by the assertions put forth by Ali et al., which postulate that the application of excessive pressure to a stiffened spine is deleterious in the treatment of patients afflicted with scoliosis (31).

Barba et al. (18) conducted a biomechanical characterization of spinal loads in individuals with scoliosis using a musculoskeletal modeling approach. The team employed a spine model with a movable thorax, which had been developed in ANYBODY software. The evaluation of the results was conducted in an upright position, with the severity of scoliosis and the type of curvature being the primary factors taken into consideration. This evaluation was performed on a population of 132 individuals afflicted with mild, moderate, and severe scoliosis. According to *in vivo* reference measurements, an increase in muscle tension was found on the convex side of the scoliosis curvature, as well as in the erector spinae and multifidus muscles. This increase was found to be gradual and proportional to the size of the scoliosis. Utilizing heat map plots, they further demonstrated that the maximum force values were predominantly observed at the spinal levels exhibiting the most substantial muscle activity. The lateral flat component of the tested force exhibited a range of values from 183 to 250 N among individuals with severe scoliosis, a decrease to 70–118 N among those with moderate curvature, and a further reduction to 27–55 N among those with mild scoliosis (18). The findings of our study align with those of Barba et al. In our study, we observed significant alterations in the tension of the muscles responsible for trunk stabilization and the pressure on the resistance head in the “1” position. The most significant alterations were observed in the Erector Spinae and Multifidis muscles. Minor alterations are indicated for the oblique abdominals (Obliquus internus abdominis) and the lumbar greater (Psoas Major). The values of muscle reaction forces in the “0” position ranged from 11.6 Newtons (N) for Obliquus Internus Abdominis to 41.7 N for Psoas Major. The values obtained for the left and right sides were analogous, with the exception of the psoas major, where a diminished result of 16.9 N was recorded on the right side. Additionally, the obliquus internus abdominis exhibited a value of 41.5 N on the right side. Conversely, in the “1” position, the range exhibited a higher variability and varied from 16.9 N for the psoas major in the “0” position. The reduced reaction force values observed in the “0” position can be attributed to the fact that the gravitational force acting on the long axis of the spine in a kneeling position with support is decreased. The authors posit that the attainment of substantial force values in the “1” active kyphotization position unequivocally substantiates the capacity to elicit substantial force reactions that propagate through the device's head. The observation of these reactions enables the customization of therapeutic interventions, encompassing the precise modulation of applied forces during exercise, thereby enhancing its efficacy. The augmented force responses exhibited by the Multifidi muscle can be attributed to its action with heightened intensity on the left side, thereby simulating the right-sided curvature of the spine in the thoracic region. This action favors the concave side of the curvature, which is consistent with the objective of the study. Consequently, these findings substantiate the advantageous impact of “PRESSIO” method on the muscular system of patients afflicted with scoliosis.

### 4.1 Study limitations and directions for further analysis

The study's limitations include its focus on a single, healthy individual in their 20s. Additionally, the head was set to replicate the right thoracic curvature of the spine. To enhance the study's generalizability, it is essential to extend it to diverse age groups and those with varying degrees of scoliosis severity.

The present study employed the static model FreePosture in the Anybody Modeling System to obtain the results of musculoskeletal loads for selected time moments of the exercise. In future work, a model based on kinematic measurements will be developed and implemented into the Anybody environment. This model will then be used to analyze the results for the entire duration of the exercise.

The subsequent pivotal undertaking entails the formulation of a customized model of the musculoskeletal system of a patient afflicted with scoliosis. This model is to be developed on the basis of data procured from medical imaging, with the kinematics of the exercise for the designated patient being recorded through the implementation of the PRESSIO method. Subsequently, simulations are to be executed within the Anybody environment.

A limitation of the present study is that the intervertebral joint reaction forces were analyzed and reported as resultant values, without decomposition into axial compression and anterior–posterior shear. Future work will include a detailed investigation of spinal load components, particularly in the context of how different configurations and positions of the DISC4SPINE resistance heads may influence the relative contributions of axial compression and shear forces.

Additionally, forthcoming studies will incorporate sensitivity analyses examining the impact of small variations in force-vector placement and intra-abdominal pressure (IAP)-related parameters. These analyses will help evaluate model robustness and the influence of these factors on spinal loading and muscle activity.

The work's pioneering nature precludes a comparison with the outcomes attained in the supported kneeling position. This is primarily due to the authors' lack of awareness of any published results pertaining to this particular therapeutic modality.

## 5 Conclusions

1. The kneeling position employed in the “PRESSIO” method is distinguished by a diminished force exerted on the spinal axis, thereby engendering conducive conditions for spinal correction.
2. Active kyphosis, as utilized in the PRESSIO method, has been shown to exert a favorable influence on the multifidus muscle, thereby augmenting its force values, particularly on the simulated concave side of the curvature. This constitutes a pivotal component in the conservative treatment of patients afflicted with scoliosis.
3. The measurement of forces applied to the upper and lower limbs provides a foundation for the precise dosage of forces within the thoracic and lumbar regions. This precision is contingent upon the angular dimensions of the curvature.

## Data Availability

The original contributions presented in the study are included in the article/Supplementary Material, further inquiries can be directed to the corresponding author.
